# Attention deficit hyperactivity disorder medications and bone mineral density of adults in the United States

**DOI:** 10.1016/j.bonr.2022.101570

**Published:** 2022-04-20

**Authors:** Michael Jeremy Lawson, Thomas A. Beltran, Carla R. Padilla, Cristóbal S. Berry-Cabán, Y. Sammy Choi

**Affiliations:** a Department of Family Medicine, Womack Army Medical Center, 2817 Reilly Road, Fort Bragg, NC 28310, USA; b Department of Research, Womack Army Medical Center, 2817 Reilly Road, Fort Bragg, NC 28310, USA

**Keywords:** APA, American Psychiatric Association, ADHD, attention deficit hyperactivity disorder, BMC, bone mineral content, BMD, bone mineral density, CDC, Centers for Disease Control and Prevention, CI, confidence interval, DXA, dual energy X-ray absorptiometry, ETS, environmental tobacco exposure, GLMs, general linear models, MEC, mobile examination center, NHANES, National Health and Nutrition Examination Survey, Bone mineral density, Attention deficit hyperactivity disorder (ADHD), Dual energy X-ray absorptiometry (DXA), Stimulant mediation, Bone

## Abstract

**Introduction:**

Several medications used to treat attention deficit hyperactivity disorder (ADHD) have been associated with diminished bone mineral density (BMD) in children. The objective of this study was to determine if evidence exists for a similar association among adults.

**Materials and methods:**

A retrospective cross-sectional analysis was conducted using data collected by the National Health Nutrition Examination Survey 2013–2018. Data from 7961 individuals aged 18 to 50, 79 of whom were taking medications to treat ADHD. Dual-energy X-ray absorptiometry scans provided measure of body composition. Linear regression models were used to examine associations between ADHD medication use and body composition.

**Results:**

Stimulant ADHD medication usage was found to be associated with decreased BMD in both the skull (−6.6%; 95% CI 5.9–7.2) (*P* < 0.05) and thoracic spine (−6.0%; 95% CI 5.1–7.0) (*P* < 0.05). No difference in BMD was seen in any other skeletal region based on stimulant ADHD medication use (*P* > 0.05). We found no evidence to suggest that duration of use affected the observed decreases in BMD, *P* > 0.05.

**Conclusion:**

This study using a nationally representative sample assessed whether stimulant medication use in adults with ADHD was associated with decreased BMD. The overall results are inconclusive. Further study is needed to better evaluate if ADHD and/or stimulant medication use is independently associated with bone health.

## Highlights

• Stimulant ADHD medication use in adults is associated with decreased bone mineral in the skull and thoracic spine.
• No other areas of axial or appendicular skeleton showed significant differences.
• There was no dose-response effect between stimulant medication use and bone mineral density.
• The overall effect of stimulant medications on adult bone health is unclear.

## 1 Introduction

Attention deficit hyperactivity disorder (ADHD), a common neuropsychiatric disorder, affects both behavior control and attention focus. ADHD is primarily diagnosed in childhood and often persists into adulthood (Barkley et al., 2002; Faraone et al., 2006; van Lieshout et al., 2013). The Centers for Disease Control and Prevention (CDC) has estimated that there are over 6.1 million or 9.4% of US children diagnosed with ADHD, and 62% of these are prescribed medications for symptomatic control (Danielson et al., 2018; CDC, 2021). While the prevalence of ADHD in US adults is unknown, estimates range between 1.1% to 4.4% (Bernardi et al., 2012; Kessler et al., 2006). These estimates, however, predate the most recent version of the American Psychiatric Association's (APA) Diagnostic and Statistical Manual of Mental Disorders and may not accurately represent current trends (APA, 2013). Recent trend analysis shows increasing diagnosis of ADHD in adults, with scant data on medication rates (Kessler et al., 2006; Chung et al., 2019). A CDC report demonstrated that in women of child-bearing age (15–44 years), ADHD medication prescriptions rose 344% between 2003 and 2015 (Anderson et al., 2018).

Both behavioral and pharmacologic therapies are used to treat ADHD, either independently or in tandem. Medications fall into two general categories, stimulant and non-stimulant. Stimulant medications such as methylphenidate, amphetamine and their derivatives are considered first-line treatments, and non-stimulants such as atomoxetine, clonidine and guanfacine are considered second-line (De Crescenzo et al., 2017; Faraone and Glatt, 2010; Thapar and Cooper, 2016). Off-label medication treatments include nortriptyline and bupropion, which are typically used for refractory cases (Thapar and Cooper, 2016). Some ADHD stimulant medications are also occasionally prescribed for other indications, such as narcolepsy, daytime somnolence, or obesity.

Stimulant ADHD medications, and to a lesser degree atomoxetine, have been associated with pediatric growth restriction (Thapar and Cooper, 2016; Schermann et al., 2018a). Several limited observational studies have demonstrated that children on stimulant ADHD medication have both diminished bone mineral density (BMD) and bone mineral content (BMC) (Feuer et al., 2016; Howard et al., 2017; Poulton et al., 2012). There is little research on the effects of ADHD medications on adult bone. We therefore examined long-term ADHD medication use and bone density in US adults.

## 2 Materials and methods

### 2.1 Data source

We performed a retrospective analysis of data collected from the National Health and Nutrition Examination Survey (NHANES), a series of ongoing cross-sectional health assessments designed to assess the health and nutrition status of non-institutionalized US residents. Each 2-year data collection cycle uses a complex multistage probability cluster design, which oversamples specific populations to obtain both adequate samples for meaningful subgroup analyses as well as more reliable parameter estimates (Botman and Moriarty, 2000). For this study, three consecutive data collection cycles covering the years 2013 to 2018 were examined.

The NHANES protocols were approved by the National Center for Health Statistics institutional review board, and written informed consent was obtained from participants 18 years and older. To mitigate the confounding effects of age-related bone loss, only adult individuals aged 18 to 50 years (n = 9559) were considered for inclusion. Consenting participants completed a home-based interview followed by a physical examination and sample collection conducted in a mobile examination center (MEC).

### 2.2 Data collection

Trained interviewers administered home-based health interviews and family questionnaires. Self-reported socio-demographic characteristics included race and ethnicity (non-Hispanic White, non-Hispanic Black, Hispanic, and other including multiracial), age and gender. Interviewers also collected detailed medical history information as well as data regarding prescription medication use. For each prescribed medication, the interviewer recorded the drug name from either the container or a pharmacy printout for verification. The duration and ICD-10-CM code for each medication was also recorded. ICD-10-CM codes were based on participant reported diagnosis, and not directly reported by a health professional or record review. Participants were pooled into two groups based on prescription medication use, those taking medications to treat ADHD, and those not on ADHD medications (controls).

During the MEC visit, medical technicians collected biospecimens and conducted physical examinations. Body mass index, blood pressure, and body composition were measured. Body composition was assessed using dual energy X-ray absorptiometry (DXA, Hologic QDR 4500A, Marlborough, MA). In addition to soft tissue measurements, DXA scans provided measure of bone mineral content, bone area, and BMD. Only individuals with valid DXA data were included in this study. Thus, pregnant women (n = 198), individuals taller than 77 in (n = 9), individuals over 450 pounds (n = 3), and those declining the scan for other reasons (n = 969) were not included. Additionally, the DXA scan of 16 participants, although completed was deemed invalid for other reasons and thus not included. Combined with the age criteria, this left 7961 participants in our sample. Due to the NHANES design, this sample represents over 120 million non-institutionalized US adults.

Serum was assessed for cotinine, a biomarker for recent tobacco use and environmental tobacco smoke exposure. Cotinine is a primary nicotine metabolite with a half-life of approximately 15 to 20 h and has been shown to better correlate with cigarette smoking than self-report (Benowitz, 1996). Participants were stratified into three groups (smokers, ETS exposure, or no ETS exposure) using previously determined racial and ethnic based cut points for identifying adult smokers. Non-Hispanic White participants were classified as smokers if their serum cotinine concentration met or exceeded 4.1 ng/mL. Cut points for non-Hispanic Blacks, Hispanics, and other races including multiracial participants, were 12.55 ng/mL, 0.92 ng/mL, and 3.63 ng/mL, respectively (Tompkins et al., 2019). Participants below these cut points but with quantifiable serum cotinine (≥0.05 ng/mL) were classified as having had ETS exposure (Kaufmann et al., 2010). Participants without measurable serum cotinine levels were classified as having no ETS exposure.

### 2.3 Statistical analysis

Weighted estimates are provided using 6-year sampling weights to account for unequal selection probabilities among participants and adjustments for differential probabilities, non-coverage, and non-response. Summary statistics for categorical variables include the number of participants as well as the weighted prevalence within each category. Weighted estimates are reported as percentages with 95% Wald confidence intervals (CI). For normally distributed continuous variables, arithmetic means are reported. For skewed continuous data, geometric means are reported. Standard errors were calculated using the Taylor series linearization method. Estimates with standard errors exceeding 30% are noted as unstable.

Pairwise comparisons were made using Rao-Scott χ2 tests of independence. Sample weighted general linear models (GLMs) were created to assess relationships between ADHD medication use and BMD using age, gender, and smoking status as covariates. Odds ratios (ORs) were estimated using logistic regression models. *P* values less than 0.05 were considered statistically significant. The Bonferroni correction was applied in cases of multiple comparisons. Missing data were treated as missing and no attempts to impute missing data were made. All analyses were conducted using SPSS Complex Samples (SPSS version 25, IBM, Chicago, IL).

## 3 Results

A total of 7961 participants between the ages of 18 and 50 years and with valid DXA data were included in our analysis. No temporal differences were noted between the data collection cycles (2013 to 2018) with respect to gender, age, race/ethnicity, income, smoking status, and ADHD medication utilization (all *P* > 0.05). The sample consisted of 51.4% men (n = 3974; 95% CI 50.2–52.5) and 48.6% women (n = 3987; 95% CI 47.5–49.8) with mean ages of 33.8 years (95% CI 33.4–34.2) and 34.4 years (95% CI 34.0–34.8), respectively. Non-Hispanic Whites comprised most of the sample (n = 2677; 58.3%; 95% CI 53.8–62.7). Hispanics comprised 19.4% (n = 2127; 95% CI 16.1–23.1). Non-Hispanic Blacks and individuals of other race/ethnic backgrounds including multiracial individuals comprised 12.1% (n = 1663; 95% CI 10.0–14.6) and 10.3% (n = 1494; 95% CI 9.0–11.7), respectively. A minority of individuals (n = 2225; 29.0%; 95% CI 26.9–31.2) were classified as active smokers. Similarly, only a fraction of participants (n = 90; 1.7%; 95% CI 1.2–2.2) were found to be taking prescription medications for ADHD. Participant characteristics are reported in Table 1.

**Table 1:** Participant characteristics (n = 7961).

Characteristic	Total		Controls		ADHD medication users	
	n	Weighted estimate (95% CI)	n	Weighted estimate (95% CI)	n	Weighted estimate (95% CI)
Gender						
Male	3974	51.4 (50.2–52.5)	3926	51.6 (50.5–52.7)	48	59.9 (46.9–71.6)
Female	3987	48.6 (47.5–49.8)	3945	48.4 (47.3–49.5)	42	40.1 (28.4–53.1)
Age category, years						
18–29	2982	36.2 (34.5–38.0)	2938	36.3 (34.6–37.9)	44	53.3 (39.3–66.9)
30–39	2325	29.6 (28.3–30.9)	2299	29.7 (28.4–31.0)	26	26.8 (15.3–42.7)
40–50	2654	34.1 (32.6–35.7)	2634	34.0 (32.5–35.6)	20	19.8 (10.6–34.1)
Race/ethnicity						
White, non-Hispanic	2677	58.3 (53.8–62.7)	2617	57.7 (53.1–62.1)	60	84.4 (76.2–90.1)
Black, non-Hispanic	2127	12.1 (10.0–14.6)	2118	12.1 (10.0–14.6)	9	3.6 (1.6–7.9)^b^
Hispanic	1663	19.4 (16.1–23.1)	1654	19.7 (16.4–23.6)	9	6.1 (3.2–11.3)
Other - Including multiracial	1494	10.3 (9.0–11.7)	1482	10.4 (9.1–11.9)	12	5.8 (2.7–12.2)^b^
Smoking status^a^						
Active smoker	2225	29.0 (26.9–31.2)	2192	28.9 (26.8–31.2)	33	40.0 (26.8–54.8)
ETS exposure	1557	18.6 (17.3–20.0)	1536	18.5 (17.2–19.9)	21	20.2 (12.2–31.6)
No ETS exposure	3804	52.4 (49.7–55.1)	3775	52.6 (49.7–55.3)	29	39.8 (28.7–52.0)

Abbreviation: CI = confidence interval; ETS = environmental tobacco exposure.

a Smoking status N = 7586 due to lack of serum cotinine data.

b Standard error >30%.

Table 2 provides prescription ADHD medications by type (stimulant, non-stimulant). Dextroamphetamine was the most commonly used prescription treatment for ADHD (n = 43; 52.2%; 95% CI 40.7–63.5). In total, stimulant based medications accounted for 88.3% (n = 79; 95% CI 77.2–94.4) of all ADHD medications in the sample. The geometric mean duration for stimulant medication use was 504 days (n = 90; 95% CI 385–659). Due to the paucity of non-stimulant medication use in the sample, mean duration of this class of medications is not reported.

**Table 2:** Drug prevalence and type among participants on ADHD medication.

Medication	n	Weighted estimate (95% CI)
Stimulants		
Amphetamine	5	3.7 (0.8–15.9)^a^
Amphetamine; Dextroamphetamine	43	52.2 (40.7–63.5)
Dextroamphetamine	1	1.7 (0.2–11.4)^a^
Lisdexamfetamine	13	18.0 (8.0–35.4)
Methylphenidate	17	11.7 (6.9–19.2)
Non-stimulants		
Atomoxetine	3	2.4 (0.6–9.1)^a^
Bupropion	7	9.5 (4.1–20.6)^a^
Clonidine	1	0.8 (0.1–5.3)^a^
Total	90	100.0 (100.0–100.0)

a Standard error >30%.

No difference in age was found between ADHD medication users and controls, *P* = 0.52. However, significant differences were found for gender (*P* = 0.02). Women were more likely to be on some form of medication compared to men (OR 1.5; 95% CI 1.1–2.0; *P* = 0.02). Despite this, men were 3 times more likely than women to be on a stimulant than a non-stimulant (OR 3.1; 95% CI 1.6–6.2; *P* < 0.01). Examinations of race/ethnicity and smoking status revealed higher rates of smoking among ADHD medication users as well as a higher proportion of non-Hispanic Whites taking the medications, both *P* < 0.001.

Using age, gender, and smoking status as covariates, GLMs were used to examine differences in the BMD of various skeletal regions between stimulant ADHD medication users and controls (see Table 3). Models indicate that users of stimulant medications exhibit decreased BMD in the skull and thoracic spine compared to individuals not taking ADHD medications (Bonferroni adjusted *P* = 0.03 and *P* < 0.01 respectively). Adjustment for age, gender, and smoking status resulted in estimated marginal means for BMD in the skull of 2.14 g/cm^2 (2.07–2.20) among stimulant users and 2.24 g/cm^2 (2.22–2.25) among individuals not taking ADHD medications. The estimated marginal mean BMD in the thoracic spine of stimulant users was 0.78 g/cm^2 (0.77–0.81) and 0.83 g/cm^2 (0.82–0.83) among individuals not taking ADHD medications. Unadjusted mean BMD values for each assessed skeletal region are reported in Table 3. GLMs were also used to assess the relationship between BMD and duration of stimulant medication use. No significant effect of duration was found among individuals on stimulant ADHD medication on BMD for either the skull or thoracic regions (both *P* > 0.05). Table 4 summarizes duration of stimulant ADHD medication use.

**Table 3:** Mean bone mineral density by location (g/cm^2^).^a^

Location	Stimulant	Control
Head	2.09 (2.02–2.16)	2.23 (2.22–2.24)
Left arm	0.77 (0.75–0.80)	0.78 (0.78–0.79)
Right arm	0.79 (0.77–0.82)	0.80 (0.80–0.81)
Left ribs	0.63 (0.61–0.66)	0.65 (0.64–0.65)
Right ribs	0.62 (0.59–0.64)	0.63 (0.63–0.64)
Thoracic spine	0.78 (0.76–0.80)	0.83 (0.82–0.83)
Lumbar spine	1.03 (1.00–1.06)	1.05 (1.04–1.05)
Pelvis	1.22 (1.18–1.26)	1.26 (1.26–1.27)
Left leg	1.17 (1.13–1.21)	1.18 (1.17–1.19)
Right leg	1.17 (1.13–1.21)	1.19 (1.18–1.20)
Total BMD	1.10 (1.07–1.12)	1.12 (1.11–1.13)

a Following Bonferroni adjustment, only the head and thoracic spine were significantly different between groups (both P < 0.05).

**Table 4:** Means by stimulant type ADHD medication use duration quartile, weighted estimate (95% CI).^a^

	Days on medication	Head (g/cm^2^)	Thoracic spine (g/cm^2^)
Sample mean	504 (385–659)	2.09 (2.05–2.13)	0.78 (0.76–0.80)
Quartile 1	58 (46–73)	2.05 (1.95–2.15)	0.79 (0.74–0.84)
Quartile 2	324 (298–354)	2.03 (1.93–2.13)	0.80 (0.79–0.81)
Quartile 3	965 (788–1181)	2.11 (2.03–2.20)	0.78 (0.76–0.81)
Quartile 4	3663 (3280–4091)	2.17 (2.09–2.25)	0.76 (0.73–0.78)

a Arithmetic means used for head and thoracic spine BMD. Geometric mean provided for days on medication due to skewed data distribution.

## 4 Discussion

Although ADHD is primarily associated with children, up to 50% of children diagnosed with ADHD continue to be significantly impaired in adulthood (Barkley et al., 2002; Faraone et al., 2006; van Lieshout et al., 2013). As recognition of ADHD in adults increases, so too does the numbers of adults treated with ADHD medications. The majority of research on the long-term effects of these medications has focused on the pediatric population where clear associations between stimulant ADHD medication use and both decreased BMD and decreased BMC of the axial and appendicular skeleton have been demonstrated (Feuer et al., 2016; Howard et al., 2017; Poulton et al., 2012).

The pathophysiology of these potential bone mass changes is not fully understood. Proposed mechanisms include increased metabolic turnover, appetite suppression resulting in lowered calcium and vitamin D intake, and in sympathetic signaling by decreasing osteoblasts and increasing osteoclasts (Rice et al., 2018). In one murine study, methylphenidate treatment resulted in smaller (femoral anterior/posterior diameter), less mineralized (decreased BMC), and weaker bones (decreased BMD) at appendicular but not axial locations. These effects were ameliorated within 5 weeks of discontinuation of treatment (Komatsu et al., 2012). In juvenile rats, osteoclast regulation was the underlying cellular pathophysiology for the adverse effects of methylphenidate on rat skeletal development, and a dose response effect was seen (Uddin et al., 2018).

Stimulant ADHD medication use might affect patients differently based upon their age since adolescent bones are rapidly growing while adult bones are primarily engaged in remodeling. In children, both appendicular and axial skeleton is affected (Feuer et al., 2016; Howard et al., 2017; Poulton et al., 2012). We, however, observed a small difference in only the axial regions of the skull and thoracic spine. Additionally, though a dose response effect would be expected for an outcome such as decreased BMD, no such relationship occurred in our study for the parts of the axial skeleton affected. This suggests a lack of association between stimulant medications and BMD. It thus remains unknown if these axial skeletal changes contribute to the incidence of bone fracture in patients undergoing ADHD pharmacotherapy.

Some observational studies have suggested a possible protective effect of stimulant ADHD medications against fractures in young adults (Schermann et al., 2018a; Ilan et al., 2018; Schermann et al., 2019; Schermann et al., 2018b). Whether this effect is due to a biological mechanism or better control of ADHD symptoms (such as inattentiveness and impulsivity) is not clear. One systematic meta-analysis supported the later and concluded that while ADHD is associated with increased risk of all physical injuries, treatment with ADHD medications reduces that risk (Ruiz-Goikoetxea et al., 2018).

One of the primary strengths of our study was the use of a large, national database to draw comparisons between regular, long-term ADHD medication use and changes in associated bone density among US adults. Because NHANES data are collected on an annual basis, this will allow researchers to analyze changes within the population over time. Additionally, we used a relatively large sample size that allowed us to adjust for demographic characteristics of age, gender, and smoking status, suggesting that our findings are not due to confounding demographic differences. This paper, however, has significant limitations. This was an observational cross-sectional study; therefore, causality cannot be determined. While we sampled across several NHANES cycles, only 79 subjects were on stimulant medications. Due to our small sample size, we were not able to perform regression models evaluating many of the possible confounders, e.g., diet, physical activity level, sleep, comorbid conditions, other medication usage, substance use, metabolism, and endocrine function. Additionally, the interpretation of our findings is further limited by types of clinical and physical data collected in each 2-year NHANES iteration.

## 5 Conclusion

This study provides the first evaluation of the effect of ADHD stimulant medications on BMD using a nationally representative sample. Statistically significant decreases in BMD were noted in the axial skeleton; no dose response effect was observed. There were no significant differences noted in the appendicular skeleton. The significance of our findings is unknown. Further research is needed to adequately assess the effect of stimulant medication and/or ADHD on bone health.

## Disclaimer

The views expressed herein are those of the authors and do not reflect the official policy of the Department of the Army, Department of Defense, or US Government.

## CRediT authorship contribution statement

Michael Jeremy Lawson: Conceptualization, Methodology, Writing - original draft.
Thomas A. Beltran: Conceptualization, Data curation, Formal analysis.
Carla R. Padilla: Conceptualization, Methodology, Writing – Review & editing.
Cristóbal S. Berry-Cabán: Conceptualization, Writing – Review & editing.
Y. Sammy Choi: Conceptualization, Supervision, Methodology, Writing – Review & editing.

## Declaration of competing interest

None.
